# Mitochondrial iron deficiency triggers cytosolic iron overload in PKAN hiPS-derived astrocytes

**DOI:** 10.1038/s41419-024-06757-9

**Published:** 2024-05-25

**Authors:** Paolo Santambrogio, Anna Cozzi, Chiara Balestrucci, Maddalena Ripamonti, Valeria Berno, Eugenia Cammarota, Andrea Stefano Moro, Sonia Levi

**Affiliations:** 1 IRCCS San Raffaele Scientific Institute, Division of Neuroscience, Milan, Italy; 2 Vita-Salute San Raffaele University, Milan, Italy; 3 IRCCS San Raffaele Scientific Institute, Advanced Light and Electron Microscopy Bioimaging Center ALEMBIC, Milan, Italy

**Keywords:** Neurodegeneration, Mechanisms of disease

## Abstract

Disease models of neurodegeneration with brain iron accumulation (NBIA) offer the possibility to explore the relationship between iron dyshomeostasis and neurodegeneration. We analyzed hiPS-derived astrocytes from PANK2-associated neurodegeneration (PKAN), an NBIA disease characterized by progressive neurodegeneration and high iron accumulation in the globus pallidus. Previous data indicated that PKAN astrocytes exhibit alterations in iron metabolism, general impairment of constitutive endosomal trafficking, mitochondrial dysfunction and acquired neurotoxic features. Here, we performed a more in-depth analysis of the interactions between endocytic vesicles and mitochondria via superresolution microscopy experiments. A significantly lower number of transferrin-enriched vesicles were in contact with mitochondria in PKAN cells than in control cells, confirming the impaired intracellular fate of cargo endosomes. The investigation of cytosolic and mitochondrial iron parameters indicated that mitochondrial iron availability was substantially lower in PKAN cells compared to that in the controls. In addition, PKAN astrocytes exhibited defects in tubulin acetylation/phosphorylation, which might be responsible for unregulated vesicular dynamics and inappropriate iron delivery to mitochondria. Thus, the impairment of iron incorporation into these organelles seems to be the cause of cell iron delocalization, resulting in cytosolic iron overload and mitochondrial iron deficiency, triggering mitochondrial dysfunction. Overall, the data elucidate the mechanism of iron accumulation in CoA deficiency, highlighting the importance of mitochondrial iron deficiency in the pathogenesis of disease.

## Introduction

Pantothenate kinase-associated neurodegeneration (PKAN, OMIM 606157) is an example of a neurologic disorder characterized by mitochondrial dysfunction and alterations in energy, iron and Ca^2+^ homeostasis [1, 2]. PKAN is caused by mutations in the pantothenate kinase 2 (PANK2) gene, one of the four PANK enzymes and the only mitochondrial form that catalyzes the first step of coenzyme A (CoA) biosynthesis [3]. PKAN is classified as one of the most common neurodegeneration with brain iron accumulation (NBIA) disorders [4, 5], which is a group of heterogeneous monogenic disorders characterized by extrapyramidal movement impairments and brain iron accumulation in the basal ganglia [6]. In particular, PKAN patients are affected by symptoms, including early-onset spastic-dystonic paraparesis with later appearance of parkinsonian features, cognitive impairment, dementia, optic nerve atrophy and retinopathy [7, 8]. The pathogenetic mechanisms of PKAN are not completely clear [9], and the available therapeutic options are only symptomatic [10].

Although iron accumulation is a hallmark of PKAN, its relationship with CoA dysfunctional biosynthesis is unclear. Several animal models for PKAN have been developed and characterized [11–15]; unfortunately, although these models exhibit several phenotypes that are common in humans, they lack severe Perls-positive iron accumulation, making these models useless for investigating the mechanism of brain iron deposition.

Previous studies on fibroblasts and induced neurons from PKAN patients highlighted the main role of mitochondria in triggering the pathological cascade [16, 17]. These studies revealed that energetic failure was associated with oxidative damage and defective heme and iron sulfur cluster (ISC) biogenesis [18], two mitochondrial iron-dependent biosynthetic processes [19]. More recently, we developed PKAN hiPSC-derived astrocytes (d-astrocytes) [20]; this model shows severe cytosolic iron accumulation, thus recapitulating an essential feature of human disease. PKAN d-astrocytes exhibit alterations in iron metabolism, mitochondrial morphology, respiratory activity, and oxidative status [20]. In addition, these astrocytes are prone to develop a stellate phenotype, thus gaining neurotoxic features and undergoing ferroptosis [20]. Interestingly, growing PKAN d-astrocytes in CoA-supplemented medium prevents iron accumulation [20], further confirming the association between iron accumulation and defective CoA biosynthesis.

CoA is a key molecule involved in several metabolic processes, such as the tricarboxylic acid cycle, fatty acid metabolism, cholesterol and ketone body biosynthesis and posttranslational protein modifications (acetylation, 4-phosphopantetheinylation, and coalation) [21]. In particular, acetyl-CoA is the acetyl donor for the acetylation [22] of α- and β-tubulin heterodimers. This posttranslational protein modification is fundamental for the control of microtubule function and stability [23]. Specifically, acetylation favors the movement of motor proteins (i.e., kinesin and dynein), which bind acetylated tubulin more effectively than nonacetylated tubulin [24, 25], thus allowing the transport of vesicles and protein complexes. Dyshomeostasis of neuronal microtubule acetylation has been observed in a variety of neurodegenerative conditions [26]. In particular, abnormal levels of tubulin acetylation are associated with axonal transport defects, plasticity deficits and pathological symptoms in disorders such as Parkinson’s, Alzheimer’s and Charcot-Marie-Tooth diseases [27–29]. Indeed, analysis of d-astrocytes undergoing constitutive exo-endocytosis, a key route for cellular iron intake, and vesicular dynamics led to the finding of a general impairment in constitutive endosomal trafficking in PKAN astrocytes [30], suggesting that the impairment of CoA biosynthesis could interfere with crucial intracellular mechanisms involved in membrane fusion and vesicular trafficking, leading to aberrant transferrin receptor-mediated iron uptake [30, 31].

Here, we validated the hypothesis that the alteration of vesicular trafficking detected in PKAN d-astrocytes could be responsible for the restriction of mitochondrial iron import, resulting in mitochondrial iron deficiency and consequent cytosolic iron overload. The impairment of vesicular trafficking is caused by a reduced level of α-tubulin acetylation, probably due to CoA deficiency. In addition, the increased β-tubulin phosphorylation further destabilized the microtubule structure/dynamics.

## Results

### Generation and characterization of d-astrocytes

**Fig. 1:**
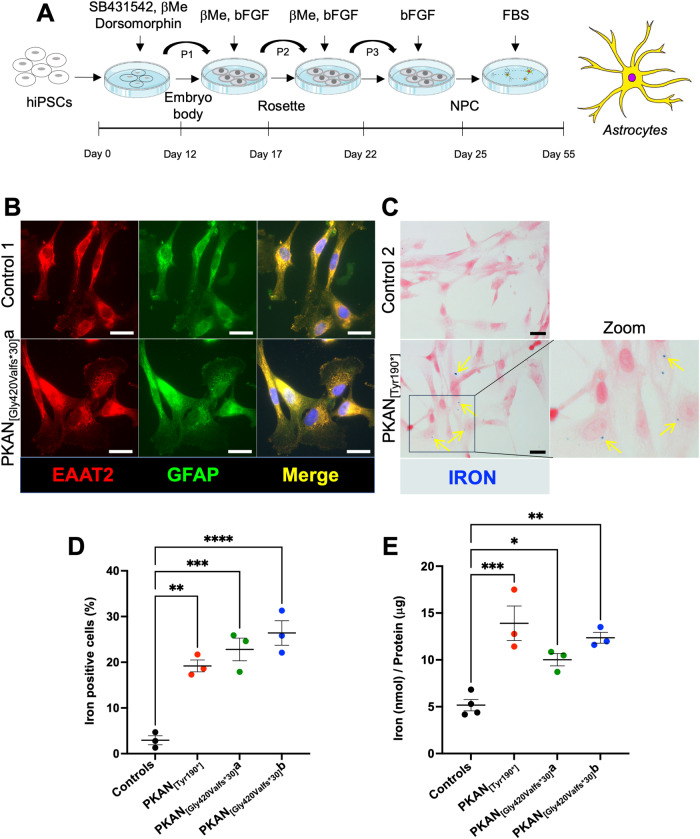
Development and characterization of d-astrocytes. **A** Graphical method representing astrocyte differentiation. NPCs were obtained by differentiating hiPSCs following a previously described method [20]. **B** Representative immunofluorescence images of d-astrocytes from one control (Control 1) and one patient (PKAN_[Gly420Valfs*30]_a) differentiated for 50 days. Astrocytes were stained with the specific markers excitatory amino acid transporter 2 (EAAT2, red) and glial fibrillary acidic protein (GFAP, green). Nuclei were stained with Hoechst (blue). Scale bars, 20 μm. **C** Representative images of d-astrocytes from one control (Control 2) and one patient (PKAN_[Tyr190*]_) differentiated for 70 days, stained with the iron-specific Perls reaction (blue), and counterstained with nuclear fast red. Arrows indicate Perls-positive granules. Scale bars, 20 μm. **D** Graph showing the percentage of cells positive for iron staining. The bars indicate the means ± SEMs of three independent experiments (one-way ANOVA followed by the Bonferroni posttest). **p < 0.01, ***p < 0.001, ****p < 0.0001. **E** Graph showing the amount of total iron in chondrocytes normalized to the total protein concentration. The bars indicate the means ± SEMs of three independent experiments (one-way ANOVA followed by the Bonferroni posttest). *p < 0.05, **p < 0.01.

Previously obtained hiPS clones [17] from three neonatal normal subjects (named control 1, 2 and 3) and three PKAN patients, two siblings carrying the PANK2 homozygous mutation c. [1259delG] (resulting in a protein with a frameshift p.[Gly420Valfs*30]a and b), and one carrying the homozygous mutation c. [569_570insA] (resulting in a premature protein stop codon p.[Tyr190*]) differentiated into neuronal precursor cells (NPCs) [17]. The cells were then further differentiated to obtain homogeneous cultures of d-astrocytes [20] following the method depicted in Fig. 1A. These new sets of differentiation were used to confirm that more than 90% of cells were positive for GFAP and EAAT2, which are astrocyte-specific markers, with no evident differences among the controls and PKAN patients, thus demonstrating that all these cells differentiated into mature d-astrocytes (Fig. 1B). Iron deposition in 70-80 day in vitro (DIV) d-astrocytes was revealed by the specific Perls reaction, in which an average of approximately 23% of the PKAN cells were iron positive, while only 3% of the control cells were mildly positive (Fig. 1C, D). Additionally, total iron, determined by the ferrozine chemical method in 50-60 DIV d-astrocytes, was significantly higher (approximately 2-3-fold) in PKAN d-astrocytes than in controls (Fig. 1E).

### Defective Transferin-loaded endosomes/mitochondrial contacts in PKAN d-astrocytes

**Fig. 2:**
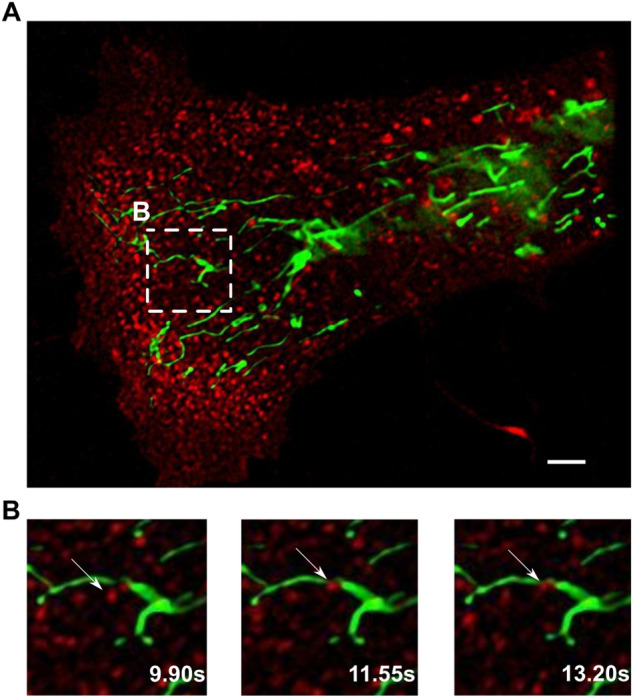
Contacts between endosomes and mitochondria in d-astrocytes. **A** Representative fluorescence microscopy image showing endocytic vesicles (labeled with the biosensor SynaptoZip, red) [47] and mitochondria (labeled with MitoTracker Green). Scale bar 5 μm. **B** Fluorescence microscopy images showing the dynamics of vesicles moving toward and interacting with mitochondria. The time frames used are reported in the images.

Our previous work aimed to investigate endosomal trafficking in PKAN d-astrocytes and indicated alterations in the dynamics of vesicular transport [30]. We performed simultaneous labeling of mitochondria and endocytic vesicles with a specific mitochondrial probe (MitoTracker) and the biosensor SynaptoZip [30] allowing live investigation of constitutive exo-endocytosis and vesicular dynamics in vitro. The results indicated that endocytic vesicles met mitochondria (an example is shown in Fig. 2). This phenomenon, called the transferrin-mediated kiss-and-run mechanism, has already been described in the literature in erythroid and epithelial cells [32–35] but not in astrocytes.

**Fig. 3:**
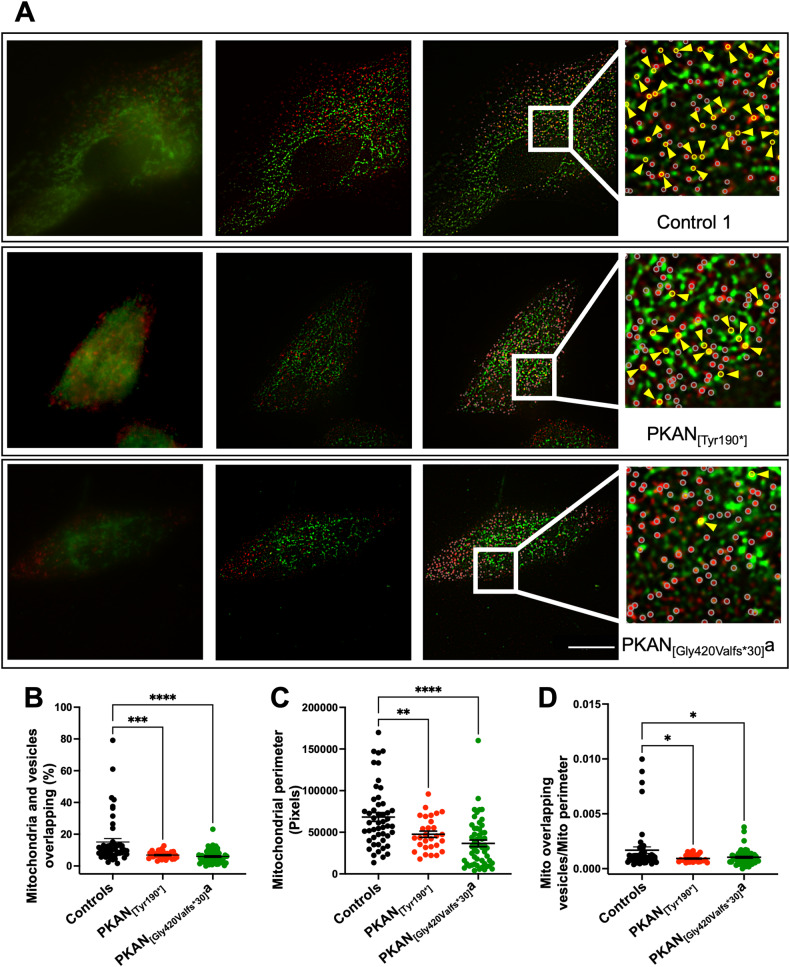
Impaired delivery of Tf-bound iron to the mitochondrial network in PKAN d-astrocytes. **A** Representative images of d-astrocytes from a control (Control 1) and two patients (PKAN [_Tyr190*_] and PKAN [_Gly420Valfs*30_]a) are shown: live-pulsed Tf-charged vesicles with fluorescent Tf (red) and TOM20 (green) staining. Epifluorescence-Hilo images (left panels) were sequentially processed with the SRRF algorithm (middle panels) and segmented for custom MATLAB routine object recognition (right panel). The positions of the vesicles are represented by empty circles (right panel and inset). The pseudocoloured yellow circles indicate vesicles overlapping (yellow arrowheads) with mitochondria, while the white circles represent all the other nonoverlapping vesicles. Scale bar = 10 μm. **B** Graph showing the percentage of transferrin-containing vesicles overlapping with mitochondria. The medians of three independent experiments were calculated (one-way ANOVA followed by the Bonferroni posttest). ***p < 0.001, ****p < 0.0001. **C** Graph showing the mean mitochondrial perimeter. The means + SEMs of three independent experiments are shown (one-way ANOVA followed by the Bonferroni posttest). **p < 0.01, ****p < 0.0001. **D** Graph showing that the Tf-containing vesicles overlapped with the mitochondria and were normalized to the mitochondrial perimeter. The means + SEMs of three independent experiments are shown (one-way ANOVA followed by the Bonferroni posttest). *p < 0.05.

To further investigate the fate of endocytosed transferrin (Tf)-bound iron, the main iron incorporation route into cells, d-astrocytes were live-pulsed for 15 min with Alexa Fluor 647-conjugated Tf to tag active endosomes that underwent receptor-mediated endocytosis. The cells were then fixed, and a specific antibody (TOM20) was used to label the mitochondrial external membrane. Images were then processed for superresolution radial fluctuations (SRRFs) to improve spatial resolution and determine the fine distribution of intracellular molecules or organelles and their potential interactions. Bona fide vesicles and mitochondria were segmented using a custom-written MATLAB routine based on their fluorescent signal, and the minimum distances of vesicles to the mitochondrial surface were measured. A significantly lower percentage (60% normalized to the mitochondrial perimeter) (Fig. 3D) of vesicles overlapped with mitochondria (minimum distance =0 pixels) in the PKAN astrocytes compared to that in the controls (Fig. 3A, B), suggesting impaired delivery of Tf-bound iron to the mitochondrial network. Moreover, these analyses revealed a significantly lower mean individual mitochondrial perimeter (Fig. 3C) in PKAN astrocytes than in control astrocytes, confirming previous findings showing altered mitochondrial morphology.

### Inefficient tubulin acetylation impairs Tf-enriched vesicle and mitochondria interactions

**Fig. 4:**
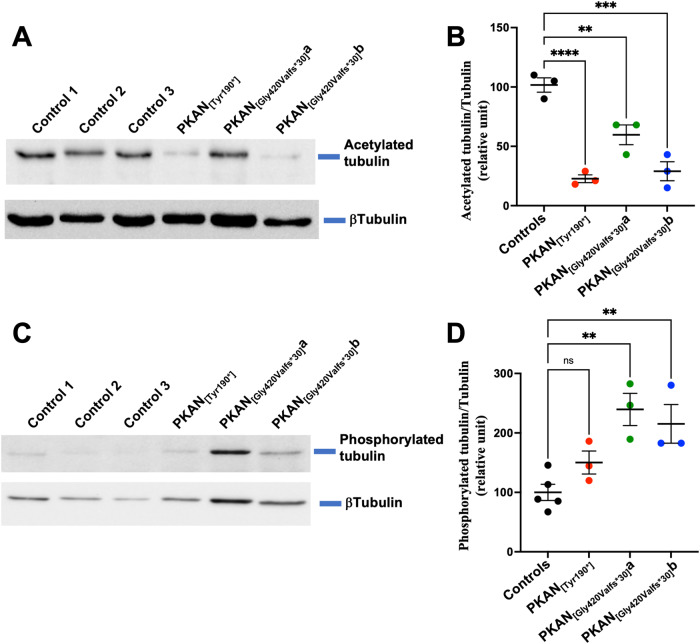
Altered tubulin acetylation and phosphorylation in PKAN d-astrocytes. **A** Western blot of soluble cell homogenates from d-astrocytes probed with anti-acetylated α-tubulin and β-tubulin as loading control. **B** The graph shows the levels of the proteins quantified via densitometry. Means + SEMs, *n* = 3 (one-way ANOVA followed by the Bonferroni posttest). **p < 0.01, ***p < 0.001. **C** Western blot of soluble cell homogenates from d-astrocytes probed with anti-phosphorylated β-tubulin and β-tubulin as loading controls. **D** The graph shows the levels of the proteins quantified via densitometry. Means + SEMs, *n* = 3 (one-way ANOVA followed by the Bonferroni posttest). **p < 0.01, ***p < 0.001.

A subpopulation of endocytic vesicles run on microtubules, a transport pathway that is functionally based on proper tubular acetylation [23]. To test whether CoA deficiency, due to genetic variation in the PKAN model, might be responsible for the alteration in vesicular trafficking, we assessed the level of α-tubulin acetylation via immunoblotting analysis. Compared with that in control astrocytes, PKAN d-astrocytes contained a reduced amount (2-4-fold) of acetylated α-tubulin (Fig. 4A, B). To support the evidence of alterations in posttranscriptional protein modification processes involved in microtubule assembly, we also analyzed the amount of phosphorylated β-tubulin. This phosphorylation impairs tubulin incorporation into microtubules [24]. We found that the level of this form was increased approximately 2-fold in PKAN d-astrocytes compared to that in controls (Fig. 4C, D). Overall, these data suggested that the alteration of this specific posttranslational modification might have consequences on iron distribution in the cell.

### Cytosolic and mitochondrial iron parameters indicate iron decompartmentalization from the mitochondria to the cytosol

**Fig. 5:**
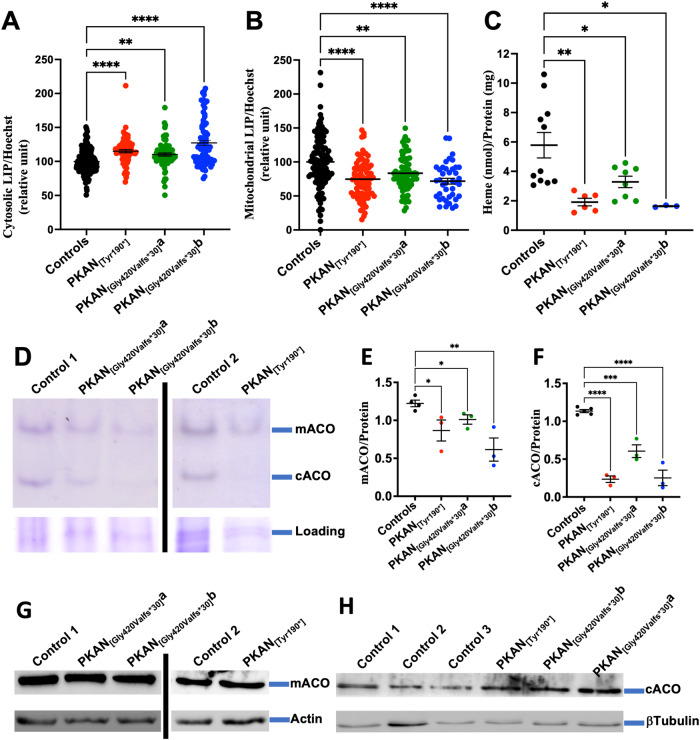
Assessment of cytosolic and mitochondrial iron parameters in d-astrocytes. **A** Evaluation of cytosolic LIP in 50 DIV d-astrocytes stained with the specific probe calcein. The graph shows the mean and SEM of three independent experiments (one-way ANOVA followed by the Bonferroni posttest). *p 0.05, ***p 0.001, ****p 0.0001. **B** The evaluation of the mitochondrial LIP in 50 DIV d-astrocytes explored by the specific probe RPA. The graph shows the mean and SEM of three independent experiments (one-way ANOVA followed by the Bonferroni posttest). ***p = 0.001, ****p = 0.0001. **C** Heme quantification in 50 DIV d-astrocytes by absorbance at 400 nm of the soluble cell lysates. The graph shows the mean and SEM of three independent experiments (one-way ANOVA followed by the Bonferroni posttest). *p 0.05, **p 0.01. **D** Representative images of in-gel enzymatic activity of mitochondrial and cytosolic aconitases (mACO and cACO, respectively) measured on 50 DIV d-astrocyte lysates. The lower part of the gel was cut and stained with Coomassie blue, and a protein band was used as a loading control (Loading). **E**, **F** Quantification of mACO and cACO enzymatic activity by densitometry. The graph shows the mean and SEM of three independent experiments (one-way ANOVA followed by the Bonferroni posttest). *p 0.05, **p 0.01, ***p 0.001, ****p 0.0001. **G**, **H** Western blot of soluble cell homogenates from 50 DIV d-astrocytes probed with anti-mACO and anti-cACO antibodies and β-actin as a loading control protein.

To explore whether the defect in the delivery of Tf-bound iron to the mitochondrial network modified the iron distribution inside the cell, we performed a broad characterization of iron metabolism in 50-70 DIV d-astrocytes, at the time point in which these cells exhibited severe iron deposition (Fig. 1C, D). In addition to the quantification of total iron (Fig. 1E), which was increased in PKAN d-astrocytes, we also explored the compartmentalization of the metal in the cells. First, we evaluated the cytosolic labile iron pool (LIP) to determine the precise index of iron status in the cytosol using the iron-sensitive fluorescent probe calcein-AM. The results indicated that a significantly higher amount of cytosolic LIP was present in all PKAN d-astrocytes compared with that in the controls (Fig. 5A). These data are also supported by the consequent expected variation in the expression of iron importers (diminished TfR1 and DMT1 + IRE), exporters (increased FPN), storage proteins (increased FtH), and ferritin cargo protein to lysosomes (diminished NCOA4) (Supplementary Fig. 1), as previously reported [20]. All these data indicate a state of cytosolic iron overload.

The amount of iron resident in the mitochondria was estimated by measuring the LIP in the organelle. In this case, the LIP was evaluated using the iron-sensitive fluorescent probe RPA. The results showed that PKAN d-astrocytes contained lower amounts of mitochondrial LIP than the controls (Fig. 5B). Furthermore, proper iron-dependent biosynthetic pathways for the production of heme and iron-sulfur clusters (ISC) were also exploited by the amount of cellular heme and the functionality of cytosolic and mitochondrial aconitases (cACO and mACO), respectively. cACO and mACO are two representative enzymes that utilize ISC as prosthetic group. Heme, measured using the spectrophotometric method was significantly lower in all PKAN d-astrocytes than in the controls (Fig. 5C). To analyze cACO and mACO, we measured their activities in-gel (Fig. 5D–F). A significant reduction in the activity of both aconitases was measured in PKAN astrocytes compared to that in control astrocytes (Fig. 5D–F). This decrease was not due to reduced protein levels since comparable amounts of mACO and slightly increased amounts of cACO were revealed by Western blot analysis (Fig. 5G, H). All these features indicate the inadequate production of heme and ISC, two of the main prosthetic groups that utilize iron in many cellular functions and biosynthetic pathways.

## Discussion

The contribution of iron to the neurodegenerative process is still under debate. It is clear that brain iron accumulation is a characteristic of many neurodegenerative pathologies, including the most widespread disorders, such as Alzheimer’s disease and Parkinson’s disease [9, 36]; however, the specific mechanism of iron deposition and its contribution to neuronal death are not yet fully understood [9]. In particular, the relationship between CoA biosynthesis defects and brain iron accumulation in PKAN disorders has not yet been elucidated [37].

**Fig. 6:**
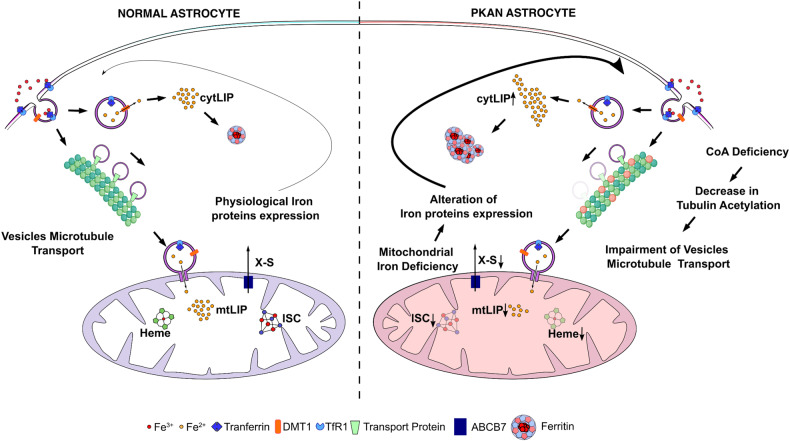
Cartoon schematic showing the proposed mechanism of iron deposition in PKAN d-astrocytes. Comparison of normal astrocytes (left) and PKAN astrocytes (right) highlighting the inefficient mitochondrial iron delivery due to impairment of tubulin acetylation caused by CoA deficiency. Iron restriction into mitochondria limits heme and ISC biosynthesis by reducing the transport of sulfur-iron-containing component (X-S) via mitochondrial transporter ABCB7. This X-S domain is necessary for sustaining the cytosolic ISC protein assembly machinery and regulating the expression of iron proteins. In the long-term, continuous iron incorporation leads to cytosolic iron overload and consequent ferritin/iron aggregation.

We took advantage of the availability of a PKAN d-astrocyte model characterized by an alteration in iron homeostasis, in which severe cytosolic iron accumulation is induced via in vitro aging (60-80 DIV) [20], allowing to follow the progression of pathological features. Here, we demonstrated that restriction of CoA causes impairment of α-tubulin acetylation, which have a negative impact on vesicular fate, inducing anomalies in the transport of Tf-loaded endocytic vesicles to mitochondria, which were detected at approximately 40-50 DIV. The consequences of these alterations include a reduced amount of iron available in mitochondria and the establishment of a mitochondrial iron deficiency status (Fig. 6). This mitochondrial iron restriction leads to impairment of iron-dependent mitochondrial pathways, such as ISC and heme cofactor biosynthesis [18, 38]. ISC and heme are essential for sustaining respiratory activity and the TCA cycle [19], which makes them fundamental for maintaining cellular energy requirements and regulating iron homeostasis [39]. A reduction in mitochondrial export of the unknown factor X-S [18] (Fig. 6), which is used to construct ISC-containing proteins, results in restriction of the availability of cytosolic ISCs, favoring the apo form of IRP1 (also named cACO) [40]. This form, by binding to the iron responsive element on the RNA messengers coding for iron- import, -export and -storage proteins, regulates their translation in a coordinated and opposite manner [41]. In particular, the stabilization of Tf receptor mRNA favors iron influx into cells. Cytosolic iron, which is not properly delivered to mitochondria, causes the degradation of ferritin cargo (NCOA4) [42], leading to ferritin accumulation that results in cytosolic Perl-positive iron aggregates, the pathognomonic sign of disease in the brains of patients with PKAN (Fig. 6). Thus, a counterintuitive effect is established: an initial iron deficiency at the mitochondrial level and its constant requirement for iron determine, in the long-term, a cellular iron overload phenotype (Fig. 6). Specifically, the mechanism of cytosolic iron accumulation is caused by mitochondrial iron deficiency, but cytosolic iron deposition is also expected to occur in the presence of general mitochondrial dysfunctions caused by different anomalies. The resulting deficit in iron cofactors might be the cause of cytosolic iron deposition, which exacerbates oxidative damage and ferroptosis [43], as we previously observed in this PKAN d-astrocyte model [20]. This causal relationship was also confirmed by the previously reported evidence that CoA administration to PKAN astrocytes at the beginning of differentiation prevents iron accumulation [20]. We cannot exclude that, given the involvement of CoA in many cellular processes, there might be also other causes that mediate the altered incorporation of iron into the mitochondrion. However, CoA deficiency has a long-range effect on iron metabolism, which is altered by factors not directly implicated in its regulation or management. This finding supports the hypothesis that mitochondrial iron metabolism is pivotal for many processes essential for cell life and that alterations in this process are very harmful. A limit of this work could be the lack of the direct measurement of the CoA amount; indeed the astrocytes differentiation method did not produce a sufficient amount of material to reach the working range of CoA determination. We infer the lower amount of CoA in patient cells since in our previous published works [17, 20] upon addition of CoA in the medium we detected an amelioration of all the observed phenotypes, including the vesicle trafficking [30].

The identification of this pathological mechanism in PKAN clarifies at least one of the relationships between CoA dyshomeostasis and iron metabolism and could be important for therapeutic purposes. Indeed, tubulin posttranslational modifications are known to modify microtubule functions [23]. Specifically, alterations in neuronal microtubule acetylation have been observed in a variety of neurodegenerative conditions, including Parkinson’s disease and Alzheimer’s disease [27, 28]. Along with their synthetic modulators, the enzymatic regulators of microtubule acetylation have also been identified [44]. Thus, a tubulin-based therapy that enhances microtubule acetylation is a potentially promising therapeutic strategy with the advantage of being helpful even for the treatment of more widespread pathologies, thus justifying the cost of drug production useful also for rare disease such as PKAN. Besides, we also highlighted the alteration of β-tubulin phosphorylation that might concur to further destabilize the microtubule structure. However, the causal relationship between the two post -translational modification of tubulin are still to be elucidated.

In conclusion, the use of this PKAN disease model allowed us i) to clarify the link between deficit of CoA and the mechanism of iron accumulation, ii) to highlight how mitochondrial iron deficiency can be the basis of an overload of cytosolic iron, corroborating the importance of iron compartmentalization, and iii) to confirm the previously described pathway for iron import to the mitochondrion in neuronal cells. In addition to clarifying the involvement of iron in the pathogenesis of CoA deficiency, this study provides new insights into iron deposition in the brain, a characteristic common to many neurodegenerative disorders, suggesting the use of iron chelators to delocalize iron inside cells.

## Materials and Methods

### Generation of human astrocytes

The hiPS clones from control and PKAN patients described in Orellana [17] were utilized to differentiate into a pure and stable population of mature astrocytes, as depicted in Fig. 1 and described in Santambrogio [20]. Briefly, NPCs were seeded onto Matrigel-coated plates and grown in DMEM-F12 supplemented with 2 mM L-glutamine (Sigma), 1% Pen/Strep (Sigma), B27 (1:200; Life Technologies), N2 (1:100; Life Technologies), and bFGF (20 ng/ml; Tebu-Bio). When the cell culture reached a confluence of approximately 60%, the medium was supplemented with 20% FBS, and the medium was changed every 2–3 days. The culture was maintained for more than 45 days to produce astrocytes with a good level of maturation.

### Immunofluorescence and immunoblotting

The cells were fixed in 4% paraformaldehyde and subjected to immunofluorescence as described in Santambrogio [20]. The antibodies used and conditions used are specified in Supplementary Table 1. Pictures were taken under a Zeiss Axio Observer Z1 fluorescence microscope equipped with a Hamamatsu EM-CCD 9100-02 camera and Volocity acquisition software. Immunoblotting was performed on soluble proteins (20 μg) separated by SDS–PAGE. The signal was visualized with an enhanced chemiluminescence (ECL) kit (GE Healthcare) and detected with a ChemiDoc MP Imaging System (Bio-Rad). A BCA protein assay (Pierce) calibrated with bovine serum albumin was used to measure the total protein.

For in-cell live recording, astrocytes at 30-40 DIV were live pulsed with the biosensor Sinaptozip to label endocytic vesicles, as described in Ripamonti [30], and with the mitochondrial marker MitoTracker Green. Video was acquired on a Widefield DeltaVision Ultra system at 3.0 fps with an environmental control unit using 60X magnification.

### Superresolution Radial Fluctuation Microscopy (SRRF)

SRRF is an open-source and high-performance analytical approach for live-cell superresolution microscopy [45]. Briefly, laser-based epifluorescence imaging with Hilo (highly inclined thin illumination) of live-pulsed Alexa Fluor 647-Transferrin and TOM20 antibody in fixed 40-50 DIV cells was performed on a Leica SR GSD 3D TIRF using a 160x TIRF objective (HC PL APO 160 X/1.4 3). Fluorescence emission was measured with an EMCCD camera (iXon Ultra 897, Andor), yielding a pixel size of 107 nm. One hundred raw images for channels were acquired with 100 ms of exposure to generate each SRRF image. SRRF images were reconstructed for each dataset using the SQUIRREL plugin in ImageJ, with the widefield images used as references. A custom-written MATLAB pipeline was constructed to measure the distances between vesicles and mitochondria.

First, we defined black and white masks for the mitochondria and vesicles. In detail, we applied the MATLAB “fibrometric” function, which enhances elongated or tubular structures for mitochondria, and the MATLAB function “imregionalmax”, which identifies the coordinates of the local maxima for the vesicles. After the two masks were unified with an OR operator, we adjusted the mask to fill the holes, slightly smooth the borders (on the scale of 5 pixels) and delete small objects (those with fewer than 100 pixels). For each vesicle, we measured the minimum distance from the mitochondrial perimeter and exported the data in Excel for statistical analysis.

### Evaluation of aconitase activity

Patient and control astrocytes were grown for 50 days in differentiation medium and then harvested, washed, and lysed as described by Orellana [17]. After centrifugation, the soluble extracts (30 μg) were added to loading buffer, loaded on PAGE gels and run at 180 V for 2.5 h at 4 °C [17]. The gel was incubated in 100 mM Tris–HCl (pH 8.0), 1 mM NADP, 5 mM MgCl2, 2.5 mM cis-aconitic acid, 1.2 mM MTT, 0.3 mM phenazine methosulfate, and 5 U/ml isocitrate dehydrogenase in the dark at 37 °C to develop aconitase activity. After image acquisition, the signal density was evaluated using ImageJ software (NIH, Bethesda, MD, USA).

### Determination of LIP

Cytosolic and mitochondrial LIP were measured using the iron-sensitive fluorescent probes calcein (Thermo Fisher Scientific) and rhodamine B-[(1,10-phenanthrolin-5-yl)-aminocarbonyl]benzyl ester (RPA) (Squarix Biotechnology), respectively. Mature astrocytes were plated in 96-well plates and incubated for 24 h. Then, for cytosolic LIP, the cells were incubated with 0.25 μM calcein-AM and 2 μg/ml Hoechst, while for mitochondrial LIP, the cells were incubated with 2 μM RPA and 2 μg/ml Hoechst. In both cases, the cells were dissolved in HBSS supplemented with 10 mM glucose, incubated for 15 min at 37 °C and then washed two or three times. Basal fluorescence was measured using Arrayscan XTI HCA Reader (Thermo Fisher Scientific) equipped with an LD Plan-NEOFLUAR 20x/0,4NA objective (Zeiss). An excitation LED (386/23 nm) was used for Hoechst, an LED (485/20 nm) was used for calcein, and a pentaband BGRFRN dichroic mirror and emission filter were used for both. An excitation LED (549/15 nm) was used for RPA with a pentaband BGRFRN dichroic mirror and single-band RS emission filters. Total calcein or RPA fluorescence was measured after the release of iron bound to the probes by the addition of the specific iron chelator PIH (final concentrations: 1 mM for calcein and 2 mM for RPA) for 30 min. The difference between total and basal fluorescence represents the cytosolic LIP. The results were normalized using Hoechst fluorescence to estimate the number of cells [20].

### Iron staining and determination of total iron and heme content

Eighty-day-old mature astrocytes were stained for iron content via the Perls reaction by incubating them for one hour in 1% potassium ferrocyanide and 1% hydrochloric acid in distilled water. The cells were counterstained with nuclear fast red (Sigma‒Aldrich). Images were taken on a Zeiss AxioImager M2m equipped with AxioCam MRc5 using a 40× objective.

Total iron was measured using the ferrozine method as described in Cairo [46]. Soluble cell homogenates were diluted in acid solution (final concentrations: 25 mM HCl, 0.1 M thioglycolic acid) and incubated for 1 hour at room temperature. After centrifugation, the clear samples were further incubated for 30 min at RT in the presence of 1 mM ferrozine. The formation of ferrozine-iron complexes was monitored by measuring the absorbance at 570 nm, and the amount of ferrozine-iron was calculated using a calibration curve obtained with a standard iron solution (Fluka, Milan, Italy).

Heme content was measured in d-astrocyte pellets after washing with PBS, dissolving in 0.25 ml of 98% formic acid and incubating for 15 min [20]. The heme content was estimated by analyzing the clear supernatant at 400 nm, with an extinction coefficient of 1.56 × 10^5^ × M^−^^1^ × cm^−^^1^. The data were normalized to the total protein content as determined by the Bio-Rad Protein Assay (Bio-Rad).

### Statistical analyses

Statistical methods to predetermine the sample size in the experiments were not employed. All experiments were performed in triplicate. Normally distributed data were analyzed with two-tailed unpaired Student’s t-test or one-way ANOVA followed by the Bonferroni posttest using GraphPad Prism. The data are reported as the means ± SEMs. A *p*-value < 0.05 was considered to indicate statistical significance.

### Supplementary information

Supplementary information

## Data Availability

The datasets generated and/or analyzed during the current study are available from the corresponding authors upon reasonable request.

## Code availability

The code generated during the current study is available from the corresponding author on reasonable request.
