# Supplementary material for: Mitochondrial iron deficiency triggers cytosolic iron overload in PKAN hiPS-derived astrocytes
Source: Cell Death Dis. 2024 May 25;15(5):361. doi: 10.1038/s41419-024-06757-9 (PMC11128011; doi:10.1038/s41419-024-06757-9)
Supplement: Supplementary file 1 — Supplementary information [file 41419_2024_6757_MOESM1_ESM.docx]

**Supplementary Information**

**Mitochondrial iron deficiency triggers neurodegeneration process in PKAN iPS-derived astrocytes.**

Santambrogio Paolo et al.

**Control 1**

**Control 2**

**PKAN_[Tyr190*]_**

**PKAN_[Gly420Valfs*30]_b**

**PKAN_[Gly420Valfs*30]_a**

**Control 3**

**TfR1**

**NCOA4**

**DMT1**

**(+IRE)**

**Actin**

**FtH**

**Actin**

**Actin**

**FPN**

**Control 2**

**PKAN_[Tyr190*]_**

**PKAN_[Gly420Valfs*30]_b**

**Control 3**

**Supplementary Figure 1. Expression of proteins involved in iron homeostasis.** Western blot of soluble cell homogenates from d-astrocytes probed with the indicated antibodies.

**FtH**

**Actin**

**Actin**

**FPN**

**Acetylated**

**tubulin**

**Phosphorylated**

**tubulin**

**βTubulin**

**cAco**

**βTubulin**

**cAco**

**mAco**

**Loading**

**Supplementary Figure 2. Uncropped western blots**

**Supplementary Table 1. Antibodies used in immunofluorescence and immunoblotting studies.**

| **Antibody-Species^a^** | **Manufacturer** | **Catalog Number** | **Application ^b^ and Dilution** |
| --- | --- | --- | --- |
| GFAP - Rb | Agilent Technologies | GA524 | IF 1:250 |
| EAAT2 - Ms | Santa Cruz Biotech. | SC-365634 | IF 1:200 |
| TOMM20-Ms | Becton Dickinson | 612278 | IF 1:500 |
| Mouse IgG Alexa488 - Dk | Imm. Sciences | IS20014 | IF 1:800 |
| Mouse IgG Alexa546 - Dk | Imm. Sciences | IS20305 | IF 1:800 |
| Rabbit IgG Alexa488 - Dk | Imm. Sciences | IS20015 | IF 1:800 |
| Rabbit IgG Alexa546 - Dk | Imm. Sciences | IS20308 | IF 1:800 |
| β-actin - Ms | Sigma–Aldrich | A5441 | WB 1:6000 |
| β-tubulin - Ms | Sigma–Aldrich | T8328 | WB 1:4000 |
| Acetilated−α-tubulin - Ms | Sigma–Aldrich | T6793 | WB 1:4000 |
| Phosphorilated−β-tubulin - Rb | Abicam | AB76286 | WB 1:9000 |
| FtH - Ms | Home made | Luzzago et al., 1986 | WB 1:500 |
| TfR1 - Mo | Zymed Laboratories | 13-6800 | WB 1:1 500 |
| FPN - Rb | Alpha Diagnostic | MTP11-S | WB 1:1 000 |
| DMT1(+IRE) - Rb | Alpha Diagnostic | NRAMP22-S | WB 1:1 000 |
| NCOA4 - Rb | Santa Cruz Biotech. | SC-28749 | WB 1:500 |
| Mouse IgG HRP - Rb | Sigma–Aldrich | A9044 | WB 1:100 000 |
| Rabbit IgG HRP - Gt | Sigma–Aldrich | A9169 | WB 1:100 000 |

^a^ Ms=mouse, Rb=rabbit, Dk=donkey, Gt=goat.

^b^ IF=immunofluorescence, WB=Western blotting (immunoblotting)
